# In-situ and airborne hyperspectral data for detecting agricultural activities in a dense forest landscape

**DOI:** 10.1016/j.dib.2023.109510

**Published:** 2023-08-20

**Authors:** C.B. Rajesh, C. V. S. S. Manohar Kumar, Sudhanshu Shekhar Jha, K.I. Ramachandran, Rama Rao Nidamanuri

**Affiliations:** a Department of Electronics and Communication Engineering, Amrita School of Engineering, Amrita Vishwa Vidyapeetham, Coimbatore 641112, India; b Department of Earth and Space Sciences, Indian Institute of Space Science and Technology, Valiamala, Thiruvananthapuram, Kerala, India; c Leipzig Institute for Meteorology, Leipzig University, Leipzig, Germany; d Department of Mechanical Engineering, Amrita School of Engineering, Amrita Vishwa Vidyapeetham, Coimbatore 641112, India

**Keywords:** Hyperspectral remote sensing, Spectral unmixing, Spectroradiometer, Mixture modelling, Target detection, Spectral signatures

## Abstract

Maintaining rich biodiversity and being a habitat and resource for humans, tropical forests are one of the most important global biomes. These forest ecosystems have been experiencing a host of unregulated anthropogenic activities including illegal tourism, and shifting cultivation. The presence of human-habitats in the restricted zones of forest ecosystems is a direct indicator of the human activities that may accelerate deterioration of forest quality by area and tree species composition. Remote sensing data have been extensively used for mapping forest types, and biophysical characterization at various spatial scales. Several remote sensing datasets from multispectral, hyperspectral and LIDAR sensors are available for developing and validating a host of methodologies for remote sensing application in forestry. However, quantifying the quality of forest stands and detecting potential threats from the sporadic and small-scale human activities requires sub-pixel level remote sensing data analysis methods such as, spectral mixture modelling. Generally, most of the studies employ pixel-level supervised learning-based analysis techniques to detect infrastructure and settlements. However, if the settlements are smaller than the ground sampling distance and are under the canopy, pixel-based techniques are not suitable. Reinvigorated with progressive availability of hyperspectral imagery, spectral mixture modelling based sub-pixel image analysis is gaining prominence in the contemporary remote sensing application development. However, there is a paucity of high-resolution hyperspectral imagery and associated ground truth spectral measurements for assessing various methodological approaches on studies related to anthropogenic activities and forest disturbance. Most of the studies have relied upon simulating and synthesising the hyperspectral imagery and its associated ground truth spectra for implementation of methods and algorithms. This article presents a distinct dataset of high-resolution hyperspectral imagery and associated ground truth spectra of various vegetable crops acquired over a tropical forest ecosystem. The dataset is valuable for research on developing new discrimination models of forest and cultivated vegetation, classification methods, spectral matching analysis techniques, and sub-pixel target detection methods.

Specifications Table

**Table:** 

Subject	Engineering
Specific subject area	Remote Sensing, Hyperspectral Remote Sensing, Forestry and Agriculture
Data format	Raw, and processed
Type of data	Hyperspectral Imagery (Raster) Spectral Signatures (In-situ)
Data collection	• Airborne visible-infrared imaging spectrometer – next generation (AVIRIS – NG) sensor was used to collect hyperspectral . • A field spectroradiometer (make and model: Spectra Vista Corporation, USA; HR-1024i) was used to collect in-situ spectral observations
Data source location	Location: Western Ghats; Country: India Subset – 1: Mudhumalai Forest Range Geographical location: 11°30.140′N 76°29.670′E Subset – 2: Naduvattam Forest Range Geographical location: 11°27.353′N 76°33.328′E
Data accessibility	Repository name: Mendeley Data Data identification number: http://doi.org/10.17632/p7n6ktjdx7.2 Direct URL to data: https://data.mendeley.com/datasets/p7n6ktjdx7
Related research article	Manohar Kumar, C. V. S. S., Nidamanuri, R. R. and Dadhwal, V. K. (2023). Subpixel Level Discrimination of Vegetable Crops in a Complex Landscape Environment, *proceedings in 2023 International Conference on Machine Intelligence for GeoAnalytics and Remote Sensing (MIGARS), IEEE Xplore.* DOI: https://doi.org/10.1109/MIGARS57353.2023.10064602

## 1 Value of the Data

• The monitoring needs of forests and farms are typically met by remote sensing imaging [1], [2], [3], [4], [5]. It is necessary to examine the forest ecosystem at the subpixel level in order to differentiate forest tree stands and cultivated plant species, as indicator of encroaching human habitats [6]. There is a lack of datasets that can distinguish between a natural forest and an agricultural crop within a dense forest ecosystem.
• In this study, we provide a hyperspectral dataset and in-situ measurements taken on common local agricultural crops in a protected, biodiversity-rich area. These datasets allow researchers to examine the direct and indirect effects of human activity on forest ecosystems.
• We can only test the existing and new mathematical methods for specific applications in limited contexts since datasets like these are so hard to come by. Using simulated dataset [7] has a serious limitation in sub-pixel image analysis since it increases the likelihood of algorithmic errors in many different kinds of applications. This distinct real hyperspectral dataset with associated ground truth are very useful for researchers for effective testing of image analysis algorithms.
• Spectral unmixing, classification, and target recognition are some of the fields that considerably benefit from hyperspectral data acquired in complex natural landscape settings.

## 2 Data Description

The entire airborne and in-situ hyperspectral data is gathered in a single folder, *‘Agriculture_Hyperspectral_Data’* and then zipped. On extracting this zip file, there are two folders, ‘Airborne_*Hyperspectral_Data*’ and *‘Insitu_Spectral_Library*’. *‘Airborne_Hyperspectral_Data*’ has two folders ‘Radiance_Data’ and ‘Reflectance_Data’. In these two folders radiance and reflectance hyperspectral imageries stored in standard ENVI *“.hdr”* format of two subsets. In the ‘*Insitu_Spectral_Library*’ two folders *‘Raw_Files’* and *‘Processed_Files. ‘Raw_Files’* folder contains two files (one is saved in *“.hdr”* format and another one is *“.txt”*) and one folder (independent spectral signatures). The ‘*Processed_Files*’ folder contains two files of spectral library saved in *“.hdr”* and *“.txt”* format. The columns number wise spectral signature details are in Table 1

**Table 1:** Spectral signatures of various vegetable, plantation crops, and a few associated forest plant species forming part of the in situ spectral library.

Column No.	Information	Crop Species	Column No.	Information	Crop Species
1	Wavelength	Nil	23	Tea 2	Tea
2	Banana 1	Banana	24	Tea 3	
3	Banana 2		25	Tea 4	
4	Banana 3		26	Tea 5	
5	Beetroot 1	Beetroot	27	Tea 6	
6	Beetroot 2		28	Tea 7	
7	Beetroot 3		29	Tea 8	
8	Bitter gourd 1	Bitter gourd	30	Eucalyptus 1	Eucalyptus
9	Bitter gourd 2		31	Eucalyptus 2	
10	Carrot 1	Carrot	32	Eucalyptus 3	
11	Carrot 2		33	Eucalyptus 4	
12	Carrot 3		34	Eucalyptus 5	
13	Carrot 4		35	Grass 3	Grass
14	Dry grass 1	Dry Grass	36	Grass 4	
15	Dry grass 2		37	Grass 5	
16	Dry grass 4		38	Pinus 1	Pinus
17	Dry grass 5		39	Pinus 2	
18	Radish 1	Radish	40	Pinus 3	
19	Radish 2		41	Silver oak 1	Silver oak
20	Radish 3		42	Silver oak 2	
21	Soil 1 carrot	Soil Type 1	43	Silver oak 3	
22	Soil 2 carrot		44	Soil 1	Soil type 2
			45	Soil 2	
			46	Soil 4	

**Fig. 1:**
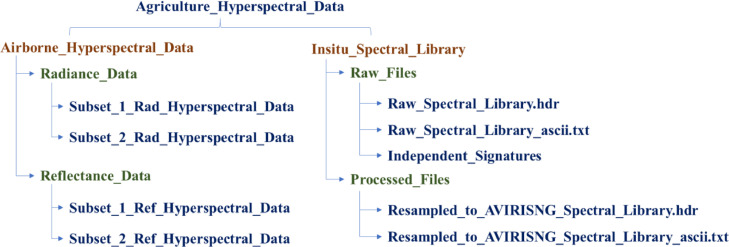
Spectral library of in-situ reflectance measurements and hyperspectral imagery datasets hierarchical structure.

File organization structure are shown in the Fig. 1.

## 3 Experimental Design, Materials and Methods

### 3.1 Experimental design

The site chosen is a federally protected forest landscape in which the maximum area is reserved for wild life sanctuaries. Human intervention due to ecotourism and unregulated transportation of forest resources lead to the forest area fragmented, and ultimately to degradation. The areas considered for imaging are topographically hilly terrain. Due to their remoteness, favourable climate, fertile soils, and availability of water, most of the forest ecosystems in the Western Ghats, India are suitable for growing different crops of commercial nature such as spices, coffee and tea. As a result, unauthorized expansion of human activities and progressive establishment of illegal human habits is a common problem. This remote sensing data acquisition experiment was undertaken to examine the potential of automated detection of human habitats using the existence of food-based vegetable crops as the proxy.

Subset – 1: When the major crops are banana and bitter-gourd, and the landscape is urban, forest and agriculture

Subset – 2: When the major crops are beetroot, carrot, and radish, and the landscape is forest, and agriculture

## 4 Data Acquisition

The airborne hyperspectral imagery was acquired using AVIRIS – NG sensor on two different forest ecosystems: Mudhumalai, and Naduvattam forest ranges with a 3.9 m spatial resolution and a 5 nm spectral resolution in the 350 - 2500 nm spectral region. Using a field spectroradiometer (Spectra Vista Corporation, HR-1024i, USA) which collects reflectance radiation at 3nm and 5nm in the electromagnetic region 350 nm to 2500 nm, we obtained point-based in-situ hyperspectral reflectance measurements of the major plantation and vegetable crops grown in the study sites. On March 20, 2018, from 08:00 to 10:30 Hrs, hyperspectral images were captured and in-situ spectral measurements were taken on March 20 and 21, 2018, from 11:00 to 13:00 Hrs in IST local time.

## 5 Data Pre-Processing Method

**Fig. 2:**
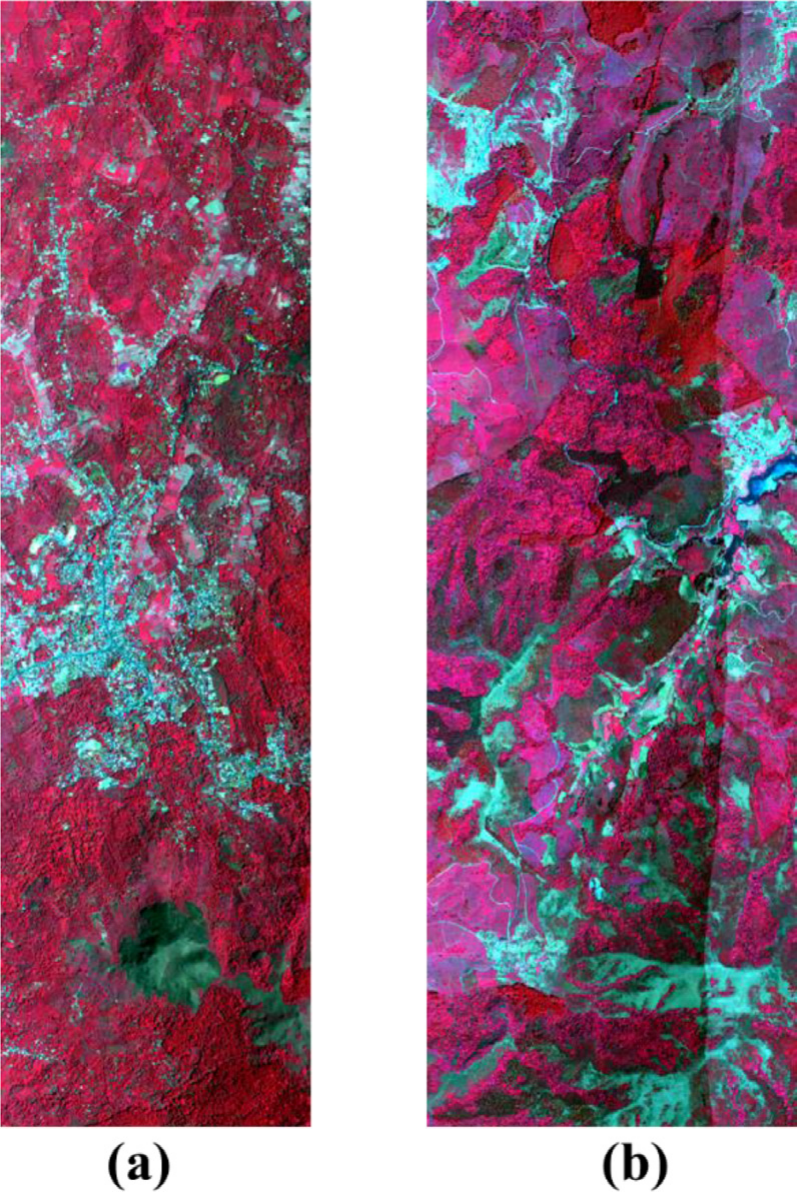
False colour composition of (a) Subset – 1 and (b) Subset – 2 acquired over Mudhumalai, and Naduvattam forest ranges of Western Ghats, India.

**Fig. 3:**
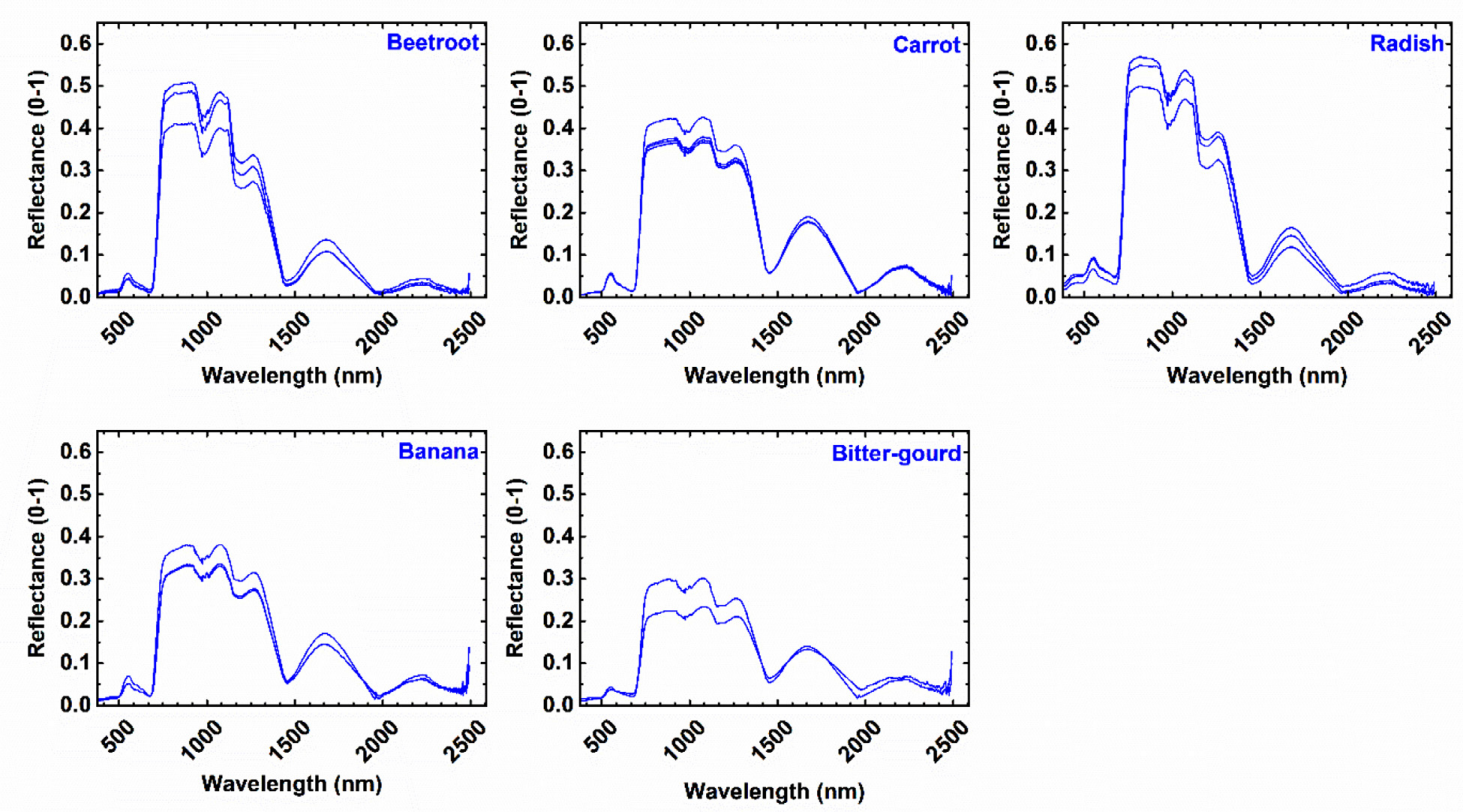
Spectral signature of vegetable crops considered.

The collected datasets of aerial hyperspectral imagery are recorded in radiance. Atmospheric correction was performed using the ATREM model [8] to transform the radiance data into surface reflectance image data. Bands of noise in the atmospherically corrected data are filtered out using the ‘*bbl’* information in the radiance header file (Figs. 2 and 3).

The spectrum measurements of reflectance made by the field spectroradiometer are catalogued as a spectral library. This spectrum library is resampled using spectral response function modelling to ensure it is compatible with hyperspectral image datasets.

## Limitations

Not applicable

## Ethics Statement

No animal or human subjects are used in the experimental set-up. The data is not collected from any social media platform.

## CRediT authorship contribution statement

**C.B. Rajesh:** Formal analysis, Writing – original draft. **C. V. S. S. Manohar Kumar:** Investigation, Writing – review & editing. **Sudhanshu Shekhar Jha:** Investigation. **K.I. Ramachandran:** Conceptualization, Methodology, Writing – review & editing. **Rama Rao Nidamanuri:** Writing – review & editing.

## Data Availability

Agriculture_Forest_Hyperspectral_Data (Original data) (Mendeley Data).
